# Importance of human microbiome: an update

**DOI:** 10.3389/frmbi.2026.1787662

**Published:** 2026-04-30

**Authors:** Maryem Wardi, Abdulmumini Baba Amin, Imane El Belghiti, Zohra Lemkhente, Ahmed Belmouden

**Affiliations:** 1 Laboratory of Cellular Biology and Molecular Genetics, Faculty of Sciences, Ibnou Zohr University, Agadir, Morocco; 2 Laboratory for Gastrointestinal Microbiology, Jiangsu Key Laboratory of Gastrointestinal Nutrition and Animal Health, National Centre for International Research on Animal Gut Nutrition, College of Animal Science and Technology, Nanjing Agricultural University, Nanjing, China; 3 Department of Animal Science, Federal University Dutse, Dutse, Jigawa, Nigeria; 4 Laboratory of Medical-Surgical, Biomedicine and Infectiology Research, Faculty of Medicine and Pharmacy, Ibnou Zohr University, Agadir, Morocco

**Keywords:** environmental health, host, human health, immunity, microbiome

## Abstract

Millions of microorganisms—including bacteria, viruses, fungi, archaea, and protists—reside on and within the human body, collectively forming the human microbiota. This complex and dynamic community plays a crucial role in modulating physiological processes, particularly the development and regulation of the immune system. Modern behaviors such as frequent washing, excessive hygiene, and widespread use of antimicrobial agents can disrupt the natural composition and functional balance of the microbiota, leading to altered immune responses and increased susceptibility to disease. In this review, we focus primarily on the bacterial component of the human microbiome. While we acknowledge the importance of viruses, fungi, archaea, and protists, these components are beyond the scope of the current review. We highlight recent advances in bacterial microbiome research that are reshaping our understanding of host–microbe interactions, immune modulation, and the health consequences of microbiota dysbiosis.

## 1 What do the terms ‘microbiota’ and ‘microbiome’ mean?

The term “microbiota” is employed to depict the diverse populations of microorganisms, encompassing both symbiotic and pathogenic, residing within and on the entire human body. These populations encompass various bacteria, viruses, fungi, archaea, and protists. The collective genetic material of all these microorganisms is referred to as the “microbiome.” It is highly individualized, varying from person to person and across different body sites. Additionally, the microbiome is dynamic and can undergo changes in response to various factors such as diet and stress. The exploration of the human microbiome began in 2007 with the launch of a project by the National Institutes of Health (NIH) aimed at identifying and characterizing the human microbiota (Peterson et al., 2009). With the advent of next-generation sequencing, this field has gained widespread attention as a promising avenue for identifying potential therapies for specific medical conditions. Numerous studies have delved into specific aspects of the microbiota, including those in the intestines, skin, and respiratory system. Moreover, researchers are increasingly uncovering the significant role of the microbiome in shaping the development of the immune system.

## 2 Skin microbiota

**Figure 1:**
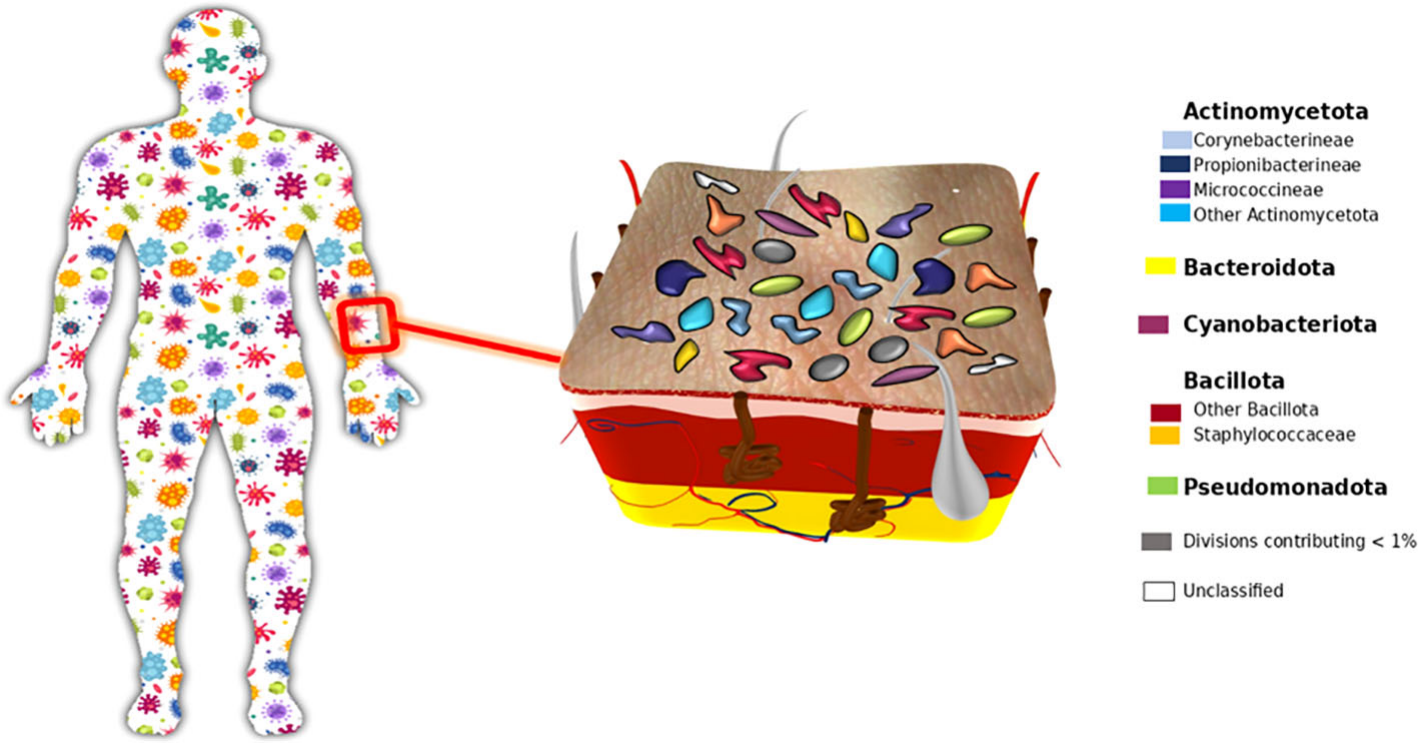
Skin microbiota.

The skin functions as the body’s outermost interface with the external environment, acting as a physical barrier to repel foreign pathogens while simultaneously hosting the commensal microbiota. The skin presents a challenging physical environment, characterized by dryness, nutrient scarcity, and acidity, all of which pose formidable obstacles to invading pathogens (Byrd et al., 2018). Within its delicate ecosystem, it houses an exceptionally dense and diverse microbial community. The skin microbiota is comprised of a vast population of bacteria, numbering around 10^12, with roughly 25% of them residing deep within the skin via hair follicles (Watanabe et al., 2024). This microbiota represents an individual’s unique microbial fingerprint and consists of millions of microorganisms, as depicted in **Figure 1** which illustrates the distribution of skin-associated microbiota across the human body. The composition is represented by major bacterial phyla, including Actinomycetota, Bacteroidota, Bacillota, Pseudomonadota, and Cyanobacteriota, demonstrating the diversity of the bacterial component of the human microbiome.

These microorganisms constitute an important defensive barrier, limiting the colonization of harmful pathogens while playing a key role in the development and regulation of the immune system (Asif et al., 2017). They are generally beneficial and non-pathogenic in immunocompetent individuals; however, under conditions such as autoimmune disorders, immunosuppression, or disruption of epithelial barriers, certain members of the microbiota may act as opportunistic pathogens.

Thanks to advancements in 16S rRNA sequencing technology, our understanding of the skin microbiome has undergone a transformative shift. Analyses reveal that Gram-positive bacteria dominate the bacterial communities on the skin. Predominant phyla observed on the skin include Actinomycetota and Bacillota. Additionally, the skin hosts Pseudomonadota and Bacteroidota (Ying et al., 2015). Furthermore, research has also identified fungal species and viruses as integral components of the skin’s microbiota (Hammoudi et al., 2021).

The composition of skin microbiota varies among individuals and can fluctuate over time. Generally, it is categorized into two groups: resident microbiota and transient microbiota. Resident microbiota, also known as normal microbiota, exhibits a consistent and stable composition over time. Transient microbiota changes depending on activities and environmental conditions.

The skin’s microbiota collaborates and acts as a defense mechanism, safeguarding the host against the intrusion of various pathogenic bacteria. Among these protective mechanisms is the production of bacteriocins, which are polypeptides with bacteriostatic or bactericidal properties. For instance, the commensal microorganism *Staphylococcus epidermidis* generates a small molecule that activates toll-like receptor 2 (TLR2), leading to the expression of antimicrobial peptides. This activation strengthens the skin’s defenses against infections (Stollery, 2006).

Furthermore, research has shown that *S. epidermidis* can shield human keratinocytes from the effects of the skin pathogen *Staphylococcus aureus in vitro* (Ying et al., 2015). This bacterium also produces phenol-soluble modulins (PSMs) that selectively inhibit skin pathogens like *S. aureus* while preserving the normal skin microbiome.

Another study has revealed that Staphylococcal species on the surface of the epidermis produce lipoteichoic acid (LTA), which has anti-inflammatory properties that inhibit skin inflammation. This inhibition specifically targets keratinocytes and is triggered by Toll-like receptor 3 (TLR3) (Stollery, 2006).

The skin microbiota is involved in metabolizing lipids and proteins while also generating bioactive molecules. In a study conducted by Brown et al., it was demonstrated that commensal coagulase-negative staphylococci (CoNS) actively influence the immune and microbial environment of the skin, fortifying its resistance against colonization or infection by opportunistic pathogens, including methicillin-resistant *Staphylococcus aureus* (MRSA) (Brown et al., 2020). CoNS produces autoinducing peptides (AIPs) that serve as effective MRSA inhibitors, thereby safeguarding the host from invasive infections. Additionally, another bacterium, *Pseudomonas aeruginosa*, triggers the production of human β-defensin-2 (Liu et al., 2002), an antimicrobial peptide with activity against Gram-negative bacteria and *Candida* spp. This multifaceted activity demonstrates the skin microbiota’s role in bolstering the body’s defenses against potential threats.

Just like all living organisms, various species within the skin microbiome engage in interactions with one another as well as with the host’s epithelial and immune cells. Under normal circumstances, this interaction confers benefits to the body by enhancing multiple aspects of barrier function. However, when this delicate balance is disrupted, it can facilitate the onset of skin diseases that lead to disarray and damage (Zhu, 2023).

Several factors influence the abundance and types of microbes present on the skin. These factors encompass diet, age, sexual maturity, anatomical location (e.g., face, trunk, extremities), skin pH, personal hygiene, and the unique characteristics of different skin areas, including the presence of hair, sebaceous glands, and moisture levels (Sinha et al., 2021):

1. Diet: It is intriguing to note that dietary factors can impact both sweat gland secretion and skin surface temperatures. Moreover, food choices can influence the sebaceous glands’ ability to excrete lipids. A high-fat diet, for instance, can alter the overall lipid composition of the skin and is associated with modifications in the microbial environment. This underscores the broad-ranging effects of our lifestyle on tissue equilibrium and the intricate interactions with our immediate surroundings (Moestrup et al., 2018).
2. Skin pH: The pH level of the skin is a crucial component of its protective system, creating an inhospitable environment for the colonization of pathogenic microorganisms. However, this barrier can be compromised in both healthy and compromised epidermis. Elevated skin pH triggers physiological responses that facilitate the attachment, growth, and invasion of microbes, especially as microbial metabolites further elevate skin pH, potentially leading to clinically apparent fungal infections (mycosis). Diabetic individuals with higher pH levels in affected skin areas are at greater risk of candidiasis. The use of mildly acidic skincare products may assist in the prevention and treatment of this type of dermatitis (Rippke et al., 2018).
3. Gender: Variations in skin microbial composition between genders may stem from physiological and anatomical differences that influence various skin characteristics, including hormone production, sweat rate, sebum production, surface pH, skin thickness, hair growth, and cosmetic usage. A recent study on human epidermis, following skin barrier disruption, revealed that women exhibited significantly greater microbial diversity on their hands than men. This diversity was linked to factors such as their less acidic skin surface and the use of cosmetics (Schommer and Gallo, 2013).
4. Moisture: Extended or continuous exposure to excessive moisture can lead to moisture-associated skin damage. In such cases, the epidermis becomes overly hydrated and more susceptible to maceration. This weakening of the skin’s resistance to mechanical forces disrupts the protective acid mantle, which acts as a barrier against irritants and microbes. This condition not only affects the skin but can also have a significant impact on an individual’s overall health. Excessive moisture not only damages the skin but also compromises the health of the skin’s microbiome, potentially creating opportunities for pathogens to enter (Parnham et al., 2020).
5. Age: A study conducted by Ying et al. (2015) identified age as one of the key factors influencing the composition of the skin bacterial community.
6. Geographical location: Urban and rural populations exhibit distinct microbial compositions, likely influenced by differences in skin conditions resulting from occupation and the substantial variation in microbial sources contributing to their microbiomes (Ying et al., 2015).

To maintain the health of our skin, it is crucial to strike a proper balance between the skin’s microbiota and adverse conditions. Practicing good hygiene is essential, but over washing or using harsh cleansers should be avoided (Byrd et al., 2018). The use of fragrances, beauty products, and scented skincare items can impact the composition of the skin microbiota. Additionally, organic solvents and detergents can harm this biodiversity. For instance, certain skincare and hygiene products can alter both molecular and bacterial diversity, as well as the dynamics and structure of molecules and bacteria on the skin (Bouslimani et al., 2019). Such disruptions can ultimately lead to skin issues such as acne or eczema.

## 3 Gut microbiota

### 3.1 Formation of microbiota in early life

**Figure 2:**
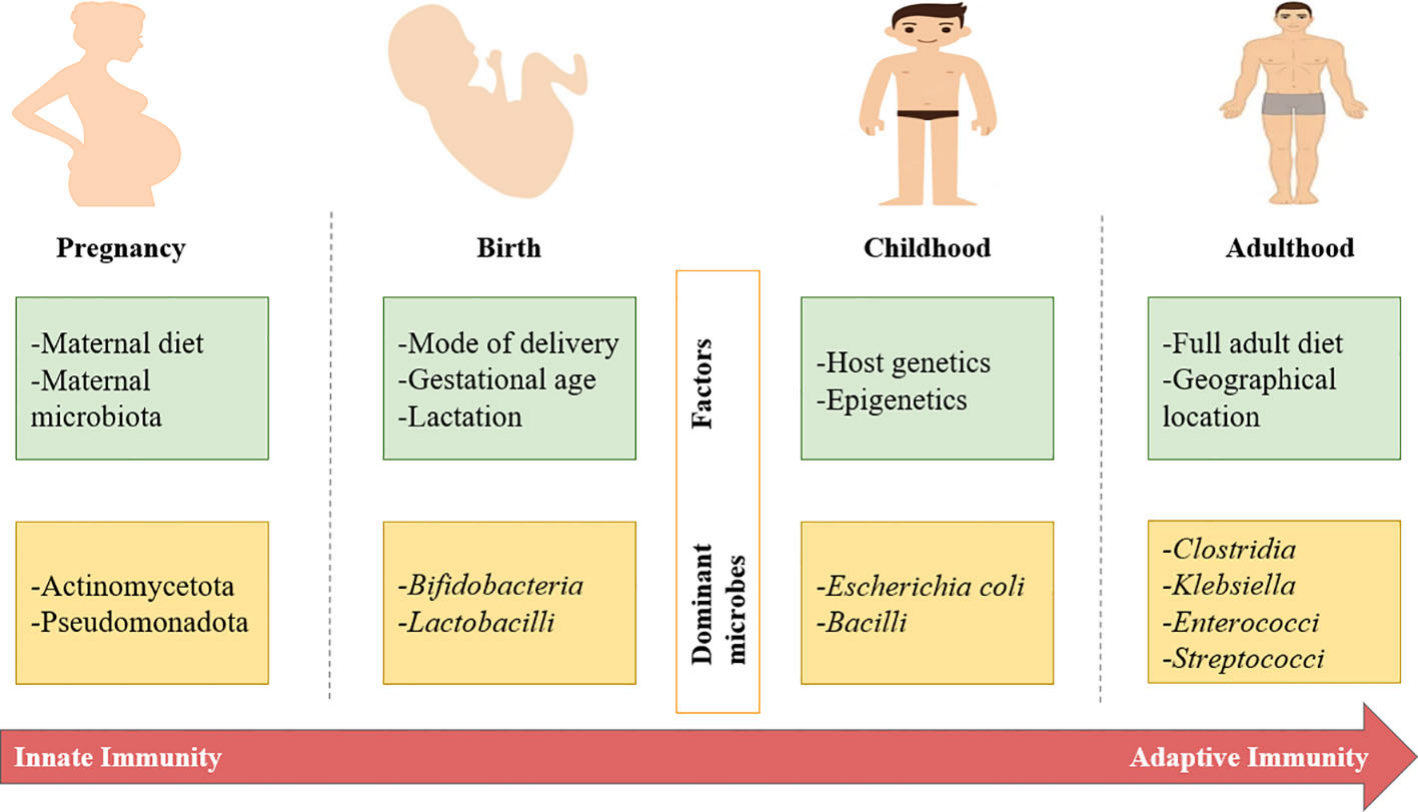
Overview of major factors and microbes that influence microbiota development from neonate to adult.

The microbiota undergoes a transformative journey from a relatively uniform state at birth to its fully mature and specialized adult form, as illustrated in **Figure 2** which summarizes the progressive development of the human gut microbiota from pregnancy to adulthood, highlighting key environmental and host-related factors at each stage. It also illustrates the shift from innate to adaptive immunity alongside changes in dominant microbial groups across the life course. During pregnancy, maternal factors such as diet and microbiota influence early microbial exposure, with dominant phyla including Actinomycetota and Pseudomonadota. At birth, factors such as mode of delivery, gestational age, and lactation shape initial colonization, characterized mainly by *Bifidobacterium* and *Lactobacillus*. In childhood, host genetics and epigenetic factors contribute to microbiota maturation, with species such as *Escherichia coli* and *Bacilli* becoming more prominent. In adulthood, diet and geographical location drive a more stable and complex microbiota, including *Clostridia*, *Klebsiella*, *Enterococci*, and *Streptococci* (Roswall et al., 2021). Numerous genetic and epigenetic factors exert their influence on the development of the microbiota. These factors include the mode of birth, instances of infections, and medication use. Among these, various studies have highlighted that the most significant factor impacting this development is the perturbation of the microbiota during early life. Such disturbances can subsequently affect its metabolic functions, potentially leading to growth deceleration (Lynch et al., 2023).

By the age of three, a complex adult microbiota typically takes shape. At this juncture, infants transition to adult diets, which play a crucial role in shaping the development of the nervous system, components of adaptive immunity, and mental health (Slykerman et al., 2019). However, it is worth noting that in certain cases, early-life exposure to microbiota disruptors like antibiotics, as well as maternal influences since newborns inherit part of their microbiota from their mothers, can disrupt microbial colonization patterns (Al Nabhani et al., 2019). In the United States, data reveals a concerning trend in antibiotic use among American children. By the age of 2, the average child has received nearly three doses of antibiotics, a number that climbs to approximately 10 doses by the age of 10, and then remains at around 10 doses by the age of 20. These statistics, while alarming, align with findings from previous nationwide surveys (Cox and Blaser, 2015). A study conducted in Denmark found that among mothers, 78% of whom had received antibiotic treatment before, during, and up to four years after pregnancy, 51% had been prescribed at least three doses of antibiotics during the study period. Notably, there was a proportional increase in the likelihood of a child developing asthma with the number of antibiotic doses the mother had taken (Kirschman et al., 2020). Combining these findings with those from other studies on the topic (Hoban et al., 2016; Pärtty et al., 2015; Sladek et al., 2022; Slykerman et al., 2019), it can be inferred that maternal antibiotic use, along with potential exposure to antibiotics through water, dairy products, and meat, as well as regional and cultural factors (Clarke et al., 2014), can impact growth, normal development, food utilization (calorie conversion into body mass), and mental health. The potential colonization of the fetus *in utero* by microbes present in the placenta and amniotic fluid remains a subject of ongoing debate, with some studies suggesting the presence of microbial signals, often dominated by Pseudomonadota. Around birth, the composition of a newborn’s microbiota is influenced by factors such as gestational age, feeding mode, and the method of delivery. As individuals progress from development to adulthood, their microbiota undergoes transformations shaped by variables such as diet, geographical location, genetics, and epigenetic factors like antibiotic use. This shift in microbiota composition coincides with the transition from innate to adaptive immunity.

### 3.2 Functions of the gut microbiota

The intestinal microbiota predominantly resides in the small intestine and the colon, with gastric acidity serving to impede colonization in the stomach. The highest microbial density is found in the colon, with the distal colon boasting a density 100 times greater than that of the proximal colon, primarily due to the increased presence of strict anaerobic bacteria (Panda et al., 2014).

The gut microbiota has garnered significant attention in numerous studies due to its profound impact on host health and its role in safeguarding against pathogens. This microbiota is multifunctional, engaging in processes such as substrate fermentation, the synthesis of vital vitamins like folate, thiamine, and biotin, and the development and maturation of the immune system. Additionally, it acts as a barrier against the colonization of pathogenic microorganisms, thereby preserving essential microbial functions within the host’s physiology. The primary bacterial groups comprising the microbiota are predominantly anaerobic (Jalili-Firoozinezhad et al., 2019), falling under the phyla Bacillota (60–80%) (including genera such as *Clostridium*, *Bacillus*, *Ruminococcus*, and *Faecalibacterium*) and Bacteroides (20–40%) (including *Bacteroides*, *Porphyromonas*, and *Prevotella*). In contrast, other groups like Pseudomonadota and Actinomycetota (such as *Bifidobacterium*, *Escherichia*, *Salmonella*, and *Helicobacter*) are present in relatively lower quantities (Jia et al., 2020).

The intestinal microbiota plays a pivotal role in various metabolic pathways, including carbohydrate metabolism, gas formation, and lipid metabolism. Carbohydrates available in the colon primarily consist of polysaccharides from grains and dietary fibers, which are broken down by fibrolytic bacteria belonging to the phyla Bacteroides (Pudlo et al., 2022) and Bacillota. These species encompass genera like *Bacteroides* (Panda et al., 2014), *Roseburia*, *Ruminoccus*, and *Eubacterium* (Bhattacharya et al., 2015). This breakdown yields short-chain fatty acids and micronutrients, such as polyphenols and vitamins, which possess antioxidant and anti-inflammatory properties.

In the context of gas metabolism, hydrogen is a byproduct of fermentation and can be eliminated via various routes, including rectally, pulmonarily, or through its recycling by colonic bacteria known as hydrogenotrophic bacteria (Sagheddu et al., 2017). These hydrogenotrophic microorganisms convert hydrogen (H_2_) into methane primarily through archaeal methanogens, as well as into acetate via acetogenic bacteria or sulfide via sulfate-reducing bacteria.

Concerning protein metabolism, several species exhibit proteolytic activity, including *Bacteroides*, *Clostridium*, *Propionibacterium*, *Streptococcus*, and *Lactobacillus* spp (Slykerman et al., 2019).

While many bacteria possess lipase activities, not all fatty acids undergo these transformations. Unsaturated fatty acids with 18 carbons are reduced by the intestinal microbiota, whereas fatty acids with 20 or 22 carbons are not metabolized. Additionally, the intestinal microbiota has the capability to convert cholesterol into coprostanol, a compound that remains unabsorbed by the intestine and is subsequently excreted in the feces (Midtvedt, 1993).

One study’s findings reveal that the gut microbiota plays a pivotal role in maintaining overall energy homeostasis, especially under cold conditions. This microbiota enhances the host’s insulin sensitivity and facilitates cold tolerance, partly by promoting the browning of white fat, leading to increased energy expenditure and fat loss (Chang et al., 2015). Moreover, the gut microbiota has an impact on behavior and anxiety regulation. It exerts control over microRNA (miRNA) expression in brain regions associated with behaviors and anxiety (Hoban et al., 2016). Additionally, gut microbes contribute to the production of colonic serotonin (5-hydroxytryptamine or 5-HT), generating metabolites that facilitate serotonin biosynthesis in the gastrointestinal tract. These metabolites influence gastrointestinal motility and hemostasis (Oliveira-Sales, 2017). Enhanced availability of carbohydrates in the hindgut promotes the synthesis of neurotransmitters in the hypothalamus, including 5-HT and dopamine, and encourages brain-derived neurotrophic factor (BDNF) expression (Gao et al., 2020). Furthermore, the gut microbiota is involved in the pathophysiology of obesity. For instance, the gut microbiota of genetically obese mice exhibits an enhanced ability to extract energy from the diet (Chang et al., 2015). Additionally, bacterial lipopolysaccharide (LPS) has been identified as a factor that triggers insulin resistance, obesity, and diabetes onset, particularly in response to a high-fat diet (Portelli, 2003). Notably, the water extract of *G. lucidum mycelium* shows promise as a potential prebiotic agent for treating obesity and its associated complications (Chang et al., 2015).

The gut microbiota also contributes to antibiotic production. Two gut bacteria, *Clostridium scindens* and *C. sordellii*, have been found to secrete antibiotics—1-acetyl-β-carboline and turbomycin A. These antibiotics appear to inhibit *C. difficile*, a bacterial pathogen responsible for causing diarrhea and colitis.

Numerous factors contribute to the variability in gut microbiota composition among individuals, including diet, age, underlying health conditions, and medical exposures such as antibiotic use.

1. Diet: Diet plays a central role in shaping the composition, diversity, and functional capacity of the gut microbiota (David et al., 2014). Specific dietary patterns, such as the Mediterranean diet, plant-based diets, or intermittent fasting, are associated with greater microbial diversity and a reduced risk of chronic diseases, including diabetes (Grigorescu et al.,2019).

For instance, the Mediterranean diet, rich in plant-based foods, olive oil, dairy, and moderate fish and poultry, promotes a healthy gut microbiome by increasing beneficial microbes such as *Faecalibacterium prausnitzii* and *Roseburia* spp., while reducing potentially harmful species (Meslier et al., 2020). These microbial shifts enhance fiber fermentation and short-chain fatty acid (SCFA) production. An enhanced version, the green Mediterranean diet, further boosts microbial diversity and metabolic activity, supporting improved body weight and cardiometabolic health (Ross et al., 2024).

Plant-based diets, rich in polyphenols and complex carbohydrates, also encourage the growth of beneficial microbes such as *Bifidobacterium* (Actinobacteriota), *Akkermansia* (Verrucomicrobiota), and *Lactobacillus* (Bacillota) (Selinger et al., 2023). Polyphenols exert prebiotic and postbiotic effects, enhancing SCFA production and providing anti-inflammatory, antimicrobial, and cardiovascular benefits (Petrova et al., 2015). Gut microbiota metabolize polyphenols into bioactive compounds that further support intestinal, metabolic, and systemic health.

Comparative studies of African and European diets have demonstrated that a fiber-rich, low-animal-protein African diet promotes the growth of *Bacteroidota* with anti-inflammatory properties, whereas the higher-protein, higher-fat European diet favors *Bacillota* proliferation (De Filippo et al., 2010). High-fiber diets, in general, increase beneficial microbes such as *Lactobacillus* and *Bifidobacterium* spp. and enhance SCFA production, supporting gut barrier integrity, appetite regulation, and metabolic health while limiting inflammation and pathogen growth (Oliver et al., 2021).

In contrast, high-protein diets, commonly adopted for athletic performance or weight loss, enrich proteolytic bacteria such as *Bacteroides* (Bacteroidota), *Clostridium* (Bacillota), and *Lacticaseibacillus* (Bacillota), which ferment amino acids into SCFAs and branched-chain fatty acids. While SCFAs confer metabolic benefits, other fermentation products—including indoles, ammonia, and hydrogen sulfide—may promote inflammation, endothelial dysfunction, and elevate colon cancer risk (Ma et al., 2017).

**Figure 3:**
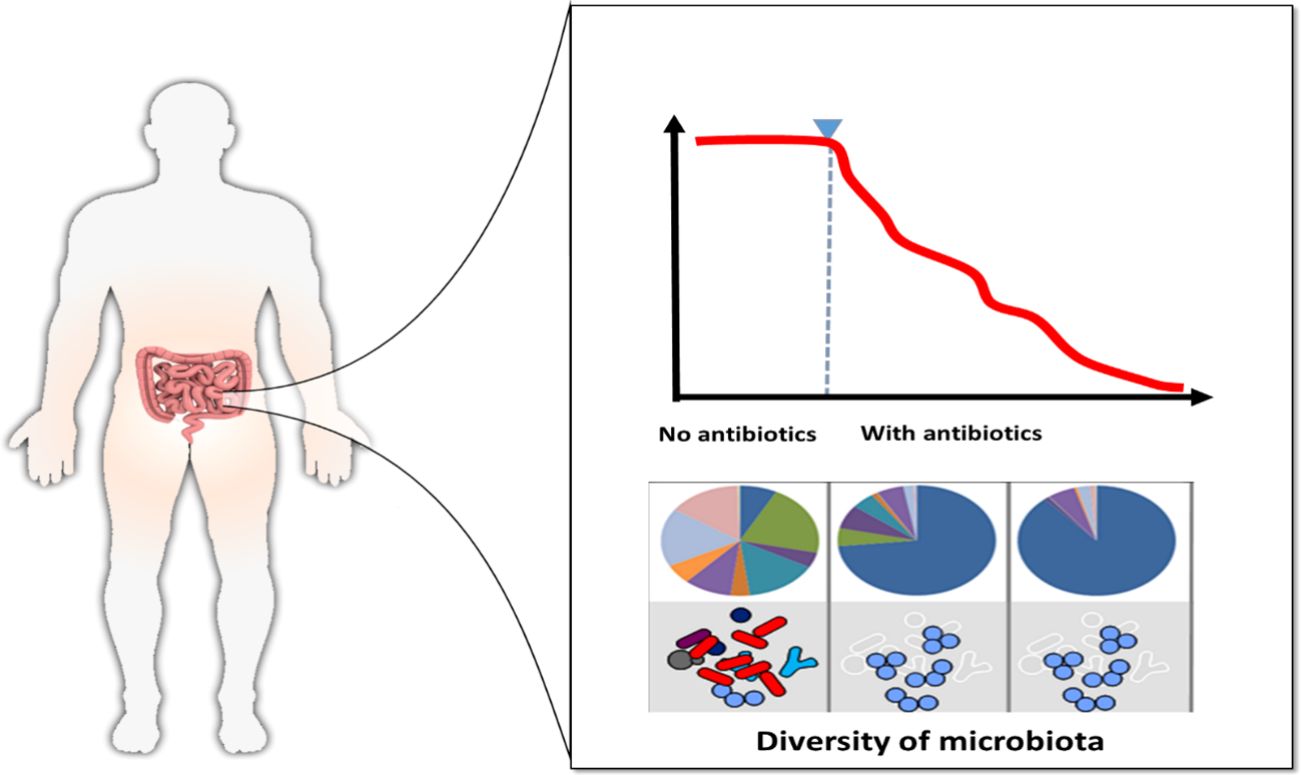
Gut microbiota before and after using antibiotics.

2. Age: Age profoundly shapes gut microbiota composition and diversity, with *Bifidobacterium* (Actinobacteriota) predominant in infancy and *Bacteroidota* and *Bacillota* dominant in adulthood. In older adults, microbial diversity declines, beneficial taxa diminish, and pro-inflammatory species rise, increasing vulnerability to infections and metabolic disorders (Gyriki et al., 2025).
3. Medical exposures: Antibiotic use is another significant determinant. While antibiotics are valuable for combatting bacterial infections, they can also lead to a substantial reduction in the diversity of the gut microbiome. They effectively target harmful bacteria but can also harm beneficial ones. Due to their overuse, many bacteria have developed resistance to antibiotics, resulting in a significant shift in the gut’s resistome and the emergence of antimicrobial resistance genes (AMR). Antibiotics not only impact infection-causing bacteria but also affect the resident microbiota, as depicted in **Figure 3**. The Figure shows a clear decline in microbial diversity following antibiotic exposure. In the absence of antibiotics, the microbiota is diverse and balanced, but with antibiotic treatment, diversity decreases significantly.

The specific effects vary depending on factors such as the type of antibiotic, dosage, and duration of use. Prolonged exposure to antibiotics can lead to the persistent presence of highly resistant bacterial strains. For instance, the administration of clindamycin has been linked to a marked and enduring increase in the levels of specific resistance genes detected in fecal DNA (Jernberg et al., 2007). This has included a substantial rise in the relative abundance of sulphonamide antibiotic resistance genes, which could contribute more effectively to resistance dissemination.

Disruption of the gut microbiota also triggers changes in local metabolomes and immune responses. For instance, fluoroquinolones and β-lactam antibiotics can significantly reduce microbial diversity by up to 25% and diminish the core phylogenetic microbiota from 29 to 12 taxa (Panda et al., 2014). Treatment with ciprofloxacin impacts approximately one-third of the bacterial taxa in the gut, resulting in reduced taxonomic richness, diversity, and evenness within the community (Jalili-Firoozinezhad et al., 2019). The depletion of butyrate-producing microbes due to antibiotic treatment can disrupt epithelial signaling through the intracellular butyrate sensor known as peroxisome proliferator–activated receptor γ (PPAR-γ). This signaling pathway serves as a homeostatic mechanism that prevents the expansion of potentially pathogenic *Escherichia* and *Salmonella* in a dysbiotic manner (Byndloss et al., 2017). Simultaneously, antibiotic administration can reduce RELMβ production and inhibit the production of interferon-γ and interleukin-17A by mucosal CD4+ T lymphocytes (Hill et al., 2010).

Alterations in the gut microbial community can also impact the bile acid pool in plasma and feces, potentially leading to significant consequences for the host (Behr et al., 2019). Conditions such as inflammatory bowel disease (IBD), Crohn’s disease, and ulcerative colitis often result from disruptions in intestinal microbes and immune system interactions. These disturbances in the gut microbiota can even contribute to an escalation of central nervous system-directed autoimmunity. The use of antibiotics in early life may have adverse effects on immune system regulation (Stanisavljević et al., 2019).

There are several additional factors that influence the composition of the intestinal microbiota. Sleep patterns, for instance, play a role in microbiota balance, with disrupted or unbalanced sleep having the potential to disturb the microbiota (Poroyko et al., 2016). Moreover, stress can elevate the risk of dysbiosis by promoting the proliferation of pathogenic bacteria. Chronic psychological stress, in particular, has been associated with a reduction in bacteria belonging to the Bacteroidota category while favoring an increase in Clostridia, which can contribute to dysbiosis and lead to infections in larger quantities (Zhang et al., 2023).

**Figure 4:**
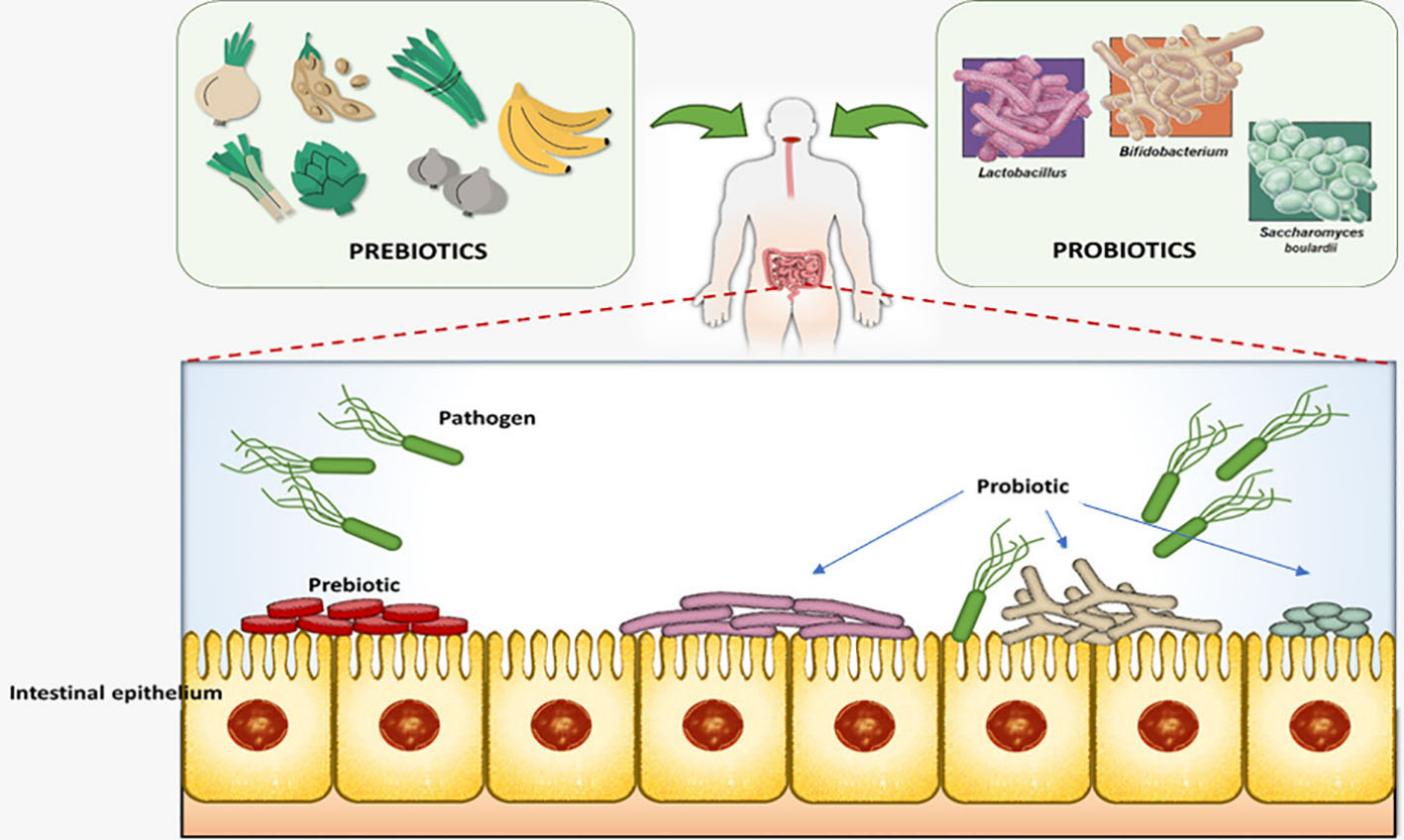
Probiotic and prebiotic in gut microbiota.

While probiotic and prebiotic compounds may contribute to intestinal microbiota modulation, their use should be evaluated on a case-by-case basis, as benefits are not consistent across all individuals (see **Figure 4**). This figure shows that Prebiotic-rich foods provide substrates stimulate the growth of beneficial gut microorganisms, while probiotics introduce beneficial bacteria such as *Lactobacillus*, *Bifidobacterium*, and *Saccharomyces boulardii* into the gastrointestinal tract. At the intestinal epithelium level, these beneficial microbes colonize the gut surface, compete with pathogenic bacteria for adhesion sites and nutrients, and contribute to strengthening the intestinal barrier.

Prebiotics refer to foods that support the growth or activity of beneficial intestinal bacteria, contributing to human health. These foods typically consist of oligosaccharides and polysaccharides, which can be found in fruits, vegetables, and breast milk. They aid in rebalancing the gut microbiome, offering protection against various aggressions, and promoting intestinal homeostasis. Prebiotics also enhance mineral absorption in the colon, improve stool consistency, and increase the frequency of bowel movements in individuals with mild constipation. These favorable effects collectively contribute to protection against colorectal cancer (Applications, 2019).

Probiotics, on the other hand, are a group of microorganisms with beneficial effects on human health. They are commonly used in the dairy industry, added to yogurts and cheeses. Probiotics prove particularly beneficial in cases of intestinal microbiota deficiency (Ji, 2023). It is essential for any probiotic to withstand stomach acidity to effectively influence the intestinal microbiota. Probiotics enhance the activity of the intestinal immune system, promote increased antibody production, and reduce the adhesion of pathogens to the intestinal wall. The current recommendation is to combine probiotics and prebiotics, creating a symbiotic effect. Probiotic and prebiotic foods stimulate the growth of various microorganisms in the intestine, each with its unique benefits. They introduce valuable functions to the gut microbial communities.

For example, a study showed that the use of *Lactobacillus rhamnosus hsryfm 1301* as a probiotic regulates the gut microbiota, subsequently improving lipid metabolism (Chen et al., 2014). Another probiotic, belonging to the *Bifidobacterium* spp., demonstrated beneficial anti-obesity effects and reduced metabolic endotoxin concentrations and intestinal inflammation (An et al., 2011). *Lactobacillus* and *Bifidobacterium* have also been effective in attenuating obesity-related comorbidities.

Regarding prebiotic foods, the consumption of cocoa flavanols, metabolized by resident microbiota, led to significant reductions in plasma triacylglycerol and C-reactive protein concentrations. Another prebiotic combination, consisting of long-chain inulin and oligofructose, exhibited positive results in reducing colitis and may serve as a primary therapy for chronic inflammatory bowel diseases (Hoentjen et al., 2005). Additionally, oligosaccharides display anti-adhesive properties, directly inhibiting the adherence of pathogens to the host epithelial cell surface (Shoaf et al., 2006).

### 3.3 The inter-individual and intra-individual variability and stability of gut microbiome

The gut microbiome contains many different microbes and varies from person to person. Even within the same individual, it can change over time, but a core group usually remains stable, helping with digestion, supporting the immune system, and protecting against harmful germs (Rosenberg, 2024). People who live in the same household tend to have more similar gut microbes because of shared diet and lifestyle, which account for over 20% of the differences, while genetics play only a minor role, explaining about 2–8% of variation (Goodrich et al., 2017; Rothschild et al., 2018). Within a single person, most microbial strains and their genes stay largely the same (less than 1% change), although the microbiome can still respond to short-term diet changes, seasonal cycles, and the timing of meals (Faith et al., 2014). For stability of the gut microbiome, many microbial strains persist long-term within an individual, showing very little change in gene content over time (Wolff et al., 2023). This stability is stronger in people with a rich baseline microbial community, making their microbiome more resilient to dietary changes and other environmental shifts. Despite this overall stability, the microbiome remains dynamic, responding in a person-specific way to environmental and lifestyle factors (Ma et al., 2024).

## 4 Gut-brain axis

The human gastrointestinal tract provides a nutrient-rich environment that supports commensal bacteria, which help the host access indigestible nutrients, produce energy, vitamins, and metabolites, and form protective biofilms that limit pathogen access (Mohajeri et al., 2018).

The gut microbiota also mediates metabolic responses to diet: dietary supplementation can alter microbial composition and function, while diet’s effect on metabolism depends on the existing microbial community rather than diet-induced changes (Chakrabarti et al., 2022).

The microbiota possesses the capability to directly or indirectly influence neuronal activity through the provision of vitamins, neurotransmitters, and neuroactive metabolic products such as short-chain fatty acids. A multitude of studies conducted in preclinical models, encompassing bacterial infections, probiotic interventions, fecal transplants, and analyses involving germ-free animals, have elucidated that the gut microbiota can impact brain function, subsequently influencing behavior (Mohajeri et al., 2018).

In recent times, the microbiota-gut-brain axis has emerged as a multidisciplinary field of study, attracting the attention of neurologists, endocrinologists, immunologists, microbiologists, and bioinformatics experts. This burgeoning area of research holds substantial promise (Chakrabarti et al., 2022).

The gut-brain axis encompasses the central, enteric, and autonomic nervous systems, along with the hypothalamic-pituitary-adrenal (HPA) axis. Within the microbiota-gut-brain axis, we find a network of gut microbes, including bacteria, viruses, fungi, and archaea, and their associated metabolites and by-products, all engaged in bidirectional communication. Despite accumulating evidence highlighting the significance of the microbiota-gut-brain axis in cognitive and mental health, human clinical studies have yet to definitively elucidate this communication process (Chakrabarti et al., 2022).

It is now evident that a microbiota-gut-brain “connectome” exists, with the extensive microbiome residing in the intestinal cavity contributing significantly to both gut behavior and the reciprocal signaling process. Furthermore, extra-enteric autonomic neurons, such as those within the sympathetic and parasympathetic nervous systems, communicate with and are influenced by intestinal microbes and macrophages (Gershon and Margolis, 2021).

The tenth cranial nerve, which extends from the brain to the abdomen, governs internal organ functions like digestion, heart rate, and respiration. The vagus nerve, composed of both efferent and afferent neurons, serves as a conduit for motor signals between the brain and organs, including intestinal cells, which are also impacted by the gut microbiota (Chakrabarti et al., 2022). Experimental evidence suggests that the microbiota can transmit signals by stimulating afferent sensory neurons of the vagus nerve through neuroimmune and neuroendocrine pathways. Another avenue of gut-brain interaction involves the HPA axis, which regulates the body’s response to stress. This complex system involves interactions between three endocrine glands: the hypothalamus, pituitary gland, and adrenal glands (Mohajeri et al., 2018). Through chemical communication with the nervous system, including both “direct” and “indirect” signal transmission, the intestinal microbiota plays a role in modulating homeostasis and behavioral patterns in its host animal (Morais et al., 2021). However, accessing the brain directly is impeded by the presence of the blood-brain barrier and various feedback mechanisms, making it challenging to determine precisely how these metabolites influence brain function (Mohajeri et al., 2018).

The human gut microbiota exerts a multifaceted influence on brain health through several mechanisms:

1. Components of bacterial structure, such as lipopolysaccharides (LPS), moderately stimulate the innate immune system. Excessive stimulation due to bacterial dysbiosis, small intestinal bacterial overgrowth, or increased intestinal permeability can lead to systemic or central nervous system inflammation (Galland, 2014). LPS, found in the outer membrane of most Gram-negative bacteria, particularly activate innate immunity across various species, from insects to humans. LPS comprises a polysaccharide region attached to the bacterial membrane via lipid A, a specific carbohydrate lipid component. Lipid A is the primary immunostimulatory element of LPS.
2. Cross-reactivity between bacterial proteins and human antigens may trigger maladaptive immune responses (Galland, 2014).
3. The gut microbiota plays a pivotal role in digestion, immune system activation, and the regulation of entero-endocrine signaling pathways. Additionally, it communicates with the central nervous system (CNS) by producing specific metabolic compounds like bile acids, short-chain fatty acids (SCFAs), glutamate (Glu), gamma-aminobutyric acid (GABA), dopamine (DA), norepinephrine (NE), serotonin (5-HT), and histamine (Dicks, 2022). Tryptophan, the sole precursor of serotonin, a critical monoamine neurotransmitter governing central neurotransmission and intestinal function, can also be converted into kynurenine, tryptamine, and indole, which regulate neuroendocrine and intestinal immune responses. The gut microbiome’s impact on tryptophan metabolism emerges as a significant factor influencing tryptophan processing.
4. Intestinal microbiota can synthesize hormones and neurotransmitters similar to those produced by humans. Bacterial receptors for these hormones affect microbial virulence and growth (Galland, 2014).
5. The vagus nerve, linking the enteric nervous system to the brain, receives immediate stimulation from intestinal bacteria, activating afferent neurons (Galland, 2014).

## 5 Respiratory system microbiota

The respiratory system harbors a diverse array of microbial communities that serve essential functions. These microorganisms act as guardians, preventing the excessive growth and spread of pathogens within the lungs. Moreover, they play a crucial role in educating the immune system, providing signals for immune development, and fostering immune tolerance (Li et al., 2024).

For instance, the composition of commensal microbiota plays a pivotal role in regulating the generation of virus-specific CD4 and CD8 T cells, as well as antibody responses when the respiratory system faces challenges like influenza virus infection. This regulation involves the proper activation of inflammasomes, resulting in the release of mature forms of IL-1β and IL-18—two host responses that are effective in protecting against infections (Brown et al., 2017). It’s worth noting that antibiotic treatment can make mice more susceptible to extensive viral replication in the lungs, underscoring the significance of the respiratory microbiota in combating infections.

Additionally, the respiratory microbiota actively contributes to the regulation of lung immunity by activating essential signaling pathways necessary for defense against infections. For example, it triggers granulocyte-macrophage colony-stimulating factor (GM-CSF) signaling and Nod2, which, in turn, enhance pathogen eradication and bolster respiratory defenses (Brown et al., 2017). Moreover, the microbiota aids in the destruction of pathogens like *Streptococcus pneumoniae* and *Staphylococcus aureus* by releasing peptidoglycan. These peptidoglycan molecules migrate to neutrophils in the bone marrow, facilitating a swift response to infections (Clarke et al., 2010).

Furthermore, the lung microbiota contributes to tolerance to allergens by promoting the expression of PD-L1. Early-life microbial colonization of the airways is critical for the healthy maturation of the neonatal immune system. This colonization prompts a temporary increase in the expression of PD-L1 by pulmonary dendritic cells (DCs), ultimately leading to the establishment of long-term tolerance to inhaled allergens (Gollwitzer et al., 2014).

The composition of the respiratory microbiota is influenced by several factors, including the method of birth, breastfeeding practices, vaccination status, dietary choices, and antibiotic usage. All of these variables have the capacity to modify the microbiota’s composition, facilitating the development of well-balanced communities that are capable of resisting excessive pathogen growth (Bosch et al., 2016).

## 6 Oral microbiota

Within the oral cavity lies the second-largest microbial community, trailing only the gut in size (Dewhirst et al., 2010). This community assumes a critical role in safeguarding the oral environment against pathogens and the development of diseases.

One of the significant functions of the oral microbiota is its regulation of nitric oxide (NO) production. NO contributes to various physiological processes, including the modulation of vasodilation, neural activity, host defense, and cellular energetics. Several bacteria in the oral cavity possess nitrate-reducing capabilities, including *Neisseria*, *Prevotella*, *Rothia*, and Actinomycetales. Notably, *Veillonella* spp has been identified as a major contributor to nitrate reduction within the oral cavity (Doel et al., 2005). The abundant presence of *Rothia* and *Neisseria* genera, for instance, appears to be advantageous for maintaining NO balance and associated indicators of cardiovascular health (Brown et al., 2017). Temporary removal of these bacteria through chlorhexidine mouth rinsing has been shown to lead to a transient increase in blood pressure (Bescos et al., 2020).

Moreover, the oral bacterium *Streptococcus salivarius* plays a crucial role in preventing oral infections. Specifically, *Streptococcus salivarius K12*, a strain of this bacterium, releases two antimicrobial peptides and is currently under evaluation as a probiotic (Doel et al., 2005).

The equilibrium of this microbiota is influenced by a combination of external and internal factors. Among these factors, the use of antibiotics stands out as a key determinant, significantly impacting the composition and functioning of the oral microbiota. This effect has been demonstrated in a study conducted by (Štšepetova et al., 2019), which revealed that amoxicillin usage leads to a reduction in the levels of *Neisseria*, *Streptococcus*, and *Veillonella*.

Additionally, smoking exerts a substantial influence on the overall makeup of the oral microbiota (Wu et al., 2016). Recent research has highlighted its consequences, which encompass shifts in the pH of oral saliva and the antimicrobial impact of harmful components within cigarette smoke.

Furthermore, various health conditions and dietary choices can bring about alterations in the composition of the oral microbiota. A range of factors, including chemotherapy, obesity, diabetes, and dietary sugar consumption, have been found to affect the oral microbiota’s composition (Štšepetova et al., 2019).

## 7 Vaginal microbiota

**Figure 5:**
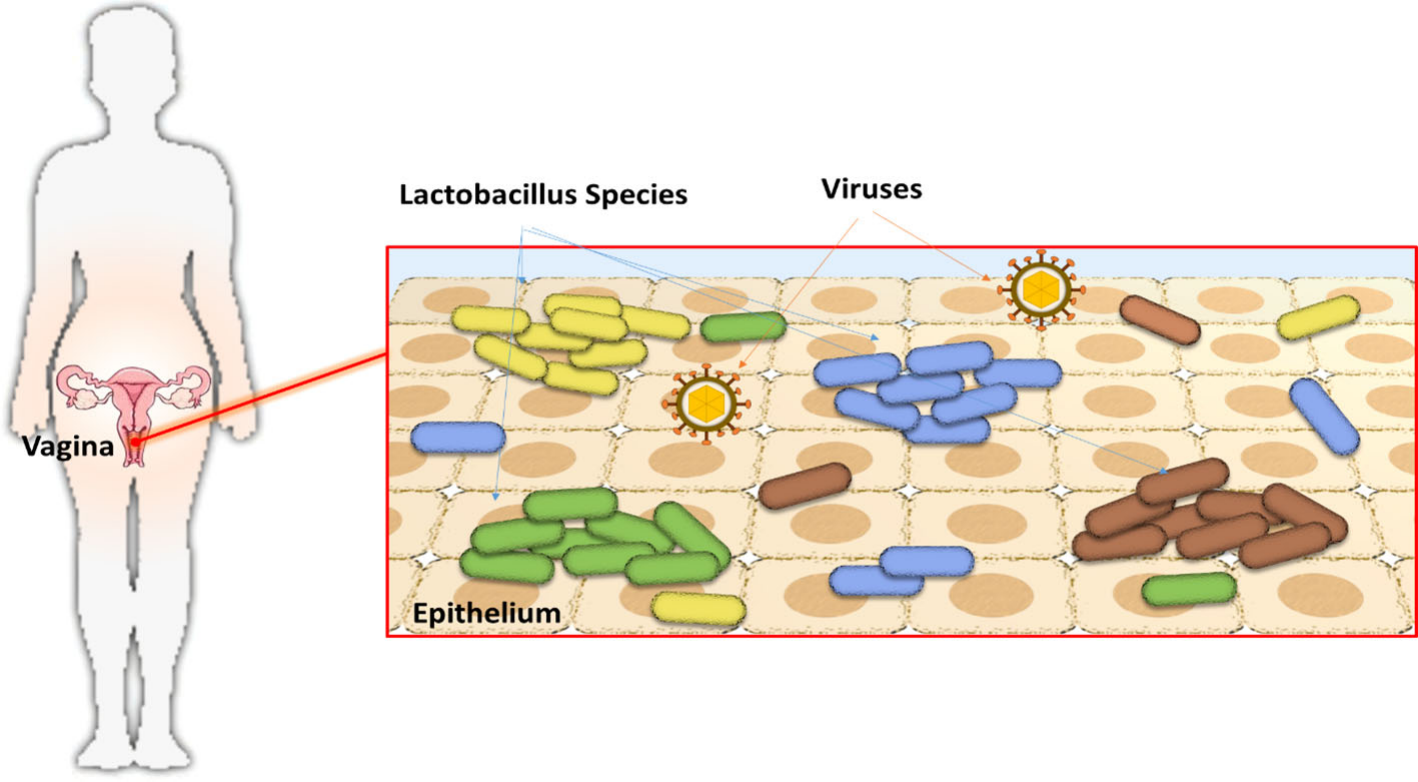
Vaginal microbiota.

The vaginal microbiome stands out as one of the most intricate ecosystems, exerting a profound impact on women’s health. It displays remarkable variability, undergoing shifts in response to diverse hormonal changes and lifestyle factors unique to each woman. Crucially, it acts as a protective shield against a range of pathogens, including urinary and sexually transmitted infections, while also playing a pivotal role in supporting successful reproduction (Ma et al., 2012). The dominant players in this microbiome are primarily strains of *Lactobacillus* spp (**Figure 5**), making them essential inhabitants of the female vaginal system.

The *Lactobacillus* species, characterized by their Gram-positive nature, thrive in anaerobic environments. Within this group, various strains of *Lactobacillus* coexist, each distinguished by their varying production of lactic acid. Lactic acid plays a pivotal role in enhancing the survival and optimizing the function of vaginal epithelial cells.

Among the most prolific *Lactobacillus* species are *L. salivarius, L. johnsonii, L. jensenii*, and *L. acidophilus*. These species possess the capability to hinder the proliferation of harmful pathogens by adhering to the walls of vaginal epithelial cells. Additionally, they demonstrate the capacity to synthesize antimicrobial substances such as bacteriocins and hydrogen peroxide (H2O2), which effectively inhibit the growth of numerous microorganisms. Furthermore, they actively contribute to the regulation of the local immune system (Petrova et al., 2015).

*Lactobacilli* are also proficient in utilizing glycogen, which is influenced by estrogen levels, thereby influencing the vaginal environment. Through a collaborative effort with vaginal epithelial cells, *lactobacilli* engage in the production of lactic acid, a crucial component responsible for maintaining an acidic pH within the vaginal ecosystem. Consequently, *Lactobacillus* species play a pivotal role in sustaining an optimal vaginal environment, promoting cleanliness, infection prevention, fertility, and immunity.

Nonetheless, it’s important to note that the composition of the vaginal microbiota is highly dynamic, influenced by factors such as age, race, physiological changes such as monthly hormonal fluctuations, and the immune system. Vaginal infections, medications, probiotics, lifestyle choices, and diet also exert significant effects on the vaginal microbiota.

1. Race/Ethnicity: Ethnicity is a substantial intrinsic factor linked with variance in community composition, with Caucasian and Asian women having a considerably higher prevalence of *Lactobacillus* spp. dominant microbiota than Hispanic and Black women (Mitra et al., 2016). A study conducted by (Ravel et al., 2011) revealed that women from 4 ethnic backgrounds (white, Asian, black, and Hispanic) have significantly different vaginal bacterial communities. The study also showed that Lactobacillus-dominated vaginal bacterial communities were detected in 80.2% and 89.7% of Asian and white women, but only 59.6% and 61.9% of Hispanic and black women, respectively.
2. Age: The vaginal microbiome is known to be influenced by age (France et al., 2022). The composition of the vaginal microbiota varies significantly over the course of a woman’s life, from infancy to the beginning of puberty, during reproduction and pregnancy, as well as during the transition to menopause and after menopause. Studies by Yoshikata et al. revealed that the vaginal microbiota of postmenopausal women had a substantially lower population of Lactobacillus and higher abundance of species responsible for bacterial vaginosis as compared to younger women. The authors concluded that age-related decline in estrogen levels in women influences *Lactobacillus* population, leading to the dominance of suboptimal species and an increase in vaginal microbiota diversity.
3. Diet: Reduced energy-adjusted betaine intake was associated with an increased risk of bacterial vaginosis. Betaine may have direct effects on the vaginal microenvironment or these effects could be mediated by the gastrointestinal microbiome (Tuddenham et al., 2019).
4. Contraceptives: Copper intrauterine devices may promote bacterial growth associated with bacterial vaginosis, while most hormonal contraceptives have little effect on the vaginal microbiota.
5. Probiotics: Probiotics and prebiotics promote the growth of beneficial microorganisms, modify the composition of the vaginal microbiota, and prevent intravaginal infections (Afifirad et al., 2022). In another study, *Lactobacilli* predominated the vaginal microbiota following two weeks of oral supplementation of probiotics (*Lactobacillus acidophilus La-14* and *Lacticaseibacillus rhamnosus HN001*) (France et al., 2022).
6. Sexual Intercourse: Unprotected vaginal contact introduces semen, an alkaline substance that momentarily raises vaginal pH, and has the potential to introduce new microbes and strains from the penile community into the vagina (France et al., 2022).

## 8 Understanding the core microbiome and criteria for defining a healthy microbial ecosystem

The core healthy microbiome is a stable and diverse community of microorganisms that supports overall health by maintaining ecological balance and promoting proper metabolic and immune function (Lloyd-Price et al., 2016). While this concept is primarily applied to the gut microbiome, other body sites and microbial groups—such as viruses, fungi, and archaea—are often understudied (Huffnagle and Noverr, 2013; Moissl-Eichinger et al., 2018; Virgin, 2014). Defining a healthy microbiome is challenging because of considerable variation between and within individuals, the dynamic fluctuations of microbial communities over time, environmental impacts, and limitations in current methodologies. To address this challenge, several approaches have been developed. One example is the Gut Microbiome Index (GMI), a novel statistical tool derived from large-scale meta-analyses, designed to evaluate individual health based on gut microbial composition. By analyzing over 5,000 fecal samples from Korean and international populations, this approach identified universal microbial signatures associated with health, distinguishing them from signatures linked to various diseases (Oh et al., 2022). Additionally, a healthy microbiome can be defined using a case-control approach, which compares healthy individuals with patients affected by specific diseases—a framework that has been applied in numerous studies (Ahmadi et al., 2025; Do et al., 2023; Manghi et al., 2025). Beyond these methods, several other measures have been proposed. Taxonomic and compositional markers are widely used to evaluate microbiome health by identifying specific microbial groups or community configurations associated with beneficial or adverse states. Higher abundances of beneficial taxa, such as *Bifidobacterium* spp. and *Akkermansia* spp., are frequently linked to favorable metabolic and immune profiles (Depommier et al., 2019; O’Callaghan and van Sinderen, 2016). At the community level, individuals can be categorized into broad assemblages known as enterotypes; for instance, the Bact2 enterotype has been associated with systemic inflammation, making its absence a potential indicator of a healthier state (Vieira-Silva et al., 2020). In the vaginal microbiome, community state type IV (CST IV) is often linked to an unhealthy balance and conditions such as bacterial vaginosis (Tamarelle et al., 2024). Beyond bacteria, non-bacterial members of the microbiome, including certain eukaryotes like *Blastocystis* spp. and some helminths, are increasingly being investigated for their potential roles in maintaining ecological balance and contributing to host health (Mpassy and Atiga, 2026). Functional and metabolic measures provide a complementary perspective for defining a healthy microbiome. These approaches evaluate the metabolic capabilities and biochemical functions of the microbiota, including the production of short-chain fatty acids such as butyrate, acetate, and propionate, which are essential for preserving gut barrier function, modulating immune responses, and supporting host metabolic regulation (Louis and Flint, 2017).

In summary, a healthy microbiome cannot be captured by a single metric and instead requires a multi-dimensional framework that considers lifestyle, geographic location, diet, age, genetics, and both taxonomic and metabolic functions.

## 9 Translational potential of microbiome science as a prognostic marker, predictor of treatment response and microbial manipulation

The microbiota is now recognized as a key component of precision medicine, with the potential to act as a biomarker for predicting treatment effectiveness and the risk of adverse effects. Growing evidence indicates that gut microbial composition and function are closely linked to host metabolic and inflammatory status. In particular, microbiota-derived metabolites play a critical role in maintaining physiological balance. Comparative studies between healthy individuals and patients with metabolic disorders have shown that beneficial metabolites, such as short-chain fatty acids and indole derivatives, are significantly reduced in disease states, whereas levels of pro-inflammatory compounds like trimethylamine N-oxide (TMAO) are elevated (Olalekan et al., 2024; Qiu et al., 2023). These alterations correlate with key clinical markers—including blood glucose, LDL cholesterol, and C-reactive protein (CRP)—highlighting the strong association between microbial dysbiosis and systemic inflammation (Li et al., 2025). Given these insights, several strategies aimed at modulating the microbiome—such as fecal microbiota transplantation, probiotics, and dietary interventions—are being explored to restore microbial balance and improve health outcomes (Ugwu et al., 2024). Beyond metabolic diseases, the microbiota also plays a crucial role in oncology. Certain microbial signatures can significantly influence the effectiveness of chemotherapy and immunotherapy. For example, high intratumoral abundances of *Clostridium* species or Gammaproteobacteria in pancreatic cancer have been associated with reduced survival and resistance to chemotherapeutic agents such as gemcitabine (Yao et al., 2026). Conversely, a higher abundance of *Akkermansia muciniphila* in the gut has been identified as a predictor of improved response to anti–PD-1 therapy in patients with advanced epithelial tumors (Derosa et al., 2022). Altogether, these findings underscore the therapeutic potential of microbiota modulation—including the development of genetically engineered bacteria and the use of microbial biomarkers—to optimize treatment responses and advance personalized patient care.

## 10 Conclusion

Microorganisms were historically seen primarily as disease-causing agents. However, thanks to technological advancements, particularly in next-generation sequencing and microbiology, our perception of the human microbiota has undergone a profound transformation. Numerous investigations have illuminated the vital significance of these microorganisms and their irreplaceable functions in maintaining human health. They play influential roles in shaping the development and maturation of various bodily systems, as well as bolstering our defenses against infections. By delving into the intricacies of this microbiome and its evolutionary dynamics, we may uncover novel avenues for the treatment of specific illnesses.
